# Levels of physical activity among a nationally representative sample of people in early old age: results of objective and self-reported assessments

**DOI:** 10.1186/1479-5868-11-58

**Published:** 2014-05-03

**Authors:** Rajna Golubic, Kathryn R Martin, Ulf Ekelund, Rebecca Hardy, Diana Kuh, Nicholas Wareham, Rachel Cooper, Soren Brage

**Affiliations:** 1 Medical Research Council Epidemiology Unit, University of Cambridge, Institute of Metabolic Science, Addenbrooke’s Hospital, Hills Road, Box 285, Cambridge CB2 0QQ, United Kingdom; 2 Medical Research Council Unit for Lifelong Health and Ageing at UCL, 33 Bedford Place, London WC1B 5JU, United Kingdom; 3 Laboratory of Epidemiology and Population Sciences, National Institute on Aging, Gateway Building, Suite 3C-309, 7201 Wisconsin Avenue, MSC 9205, Bethesda, MD 20892-9205, USA; 4 Institute of Applied Health Sciences, School of Medicine and Dentistry, The University of Aberdeen, Polwarth Building, Foresterhill, Aberdeen AB25 2ZD, United Kingdom; 5 Department of Sport Medicine, Norwegian School of Sport Sciences, PO 4014 Ullevål Stadion, 0806 Oslo, Norway

**Keywords:** Physical activity, Sedentary behaviour, Physical activity questionnaire, Combined sensing, Birth cohort, Old age

## Abstract

**Background:**

Detailed assessment of physical activity (PA) in older adults is required to comprehensively describe habitual PA-levels in this growing population segment. Current evidence of population PA-levels is predominantly based on self-report.

**Methods:**

We examined PA and sedentary behaviour in a nationally representative sample of British people aged 60–64, using individually-calibrated combined heart-rate and movement sensing and a validated questionnaire (EPAQ2), and the socio-demographic and behavioural factors that may explain between-individual variation in PA.

**Results:**

Between 2006–2010, 2224 participants completed EPAQ2 capturing the past year’s activity in four domains (leisure, work, transportation and domestic life) and 1787 participants provided 2–5 days of combined-sensing data. According to objective estimates, median(IQR) physical activity energy expenditure (PAEE) was 33.5 (25.3-42.2) and 35.5 (26.6- 47.3) kJ/kg/day for women and men, respectively. Median (IQR) time spent in moderate-to-vigorous PA (MVPA; >3MET), light-intensity PA (1.5-3 MET) and sedentary (<1.5 MET) was 26.0 (12.3-48.1) min/day, 5.4 (4.2-6.7) h/day and 18.0 (16.6-19.4) h/day, respectively, in women; and 41.0 (18.8-73.0) min/day, 5.2 (4.0-6.5) h/day and 17.9 (16.3-19.4) h/day in men. PAEE and time spent in MVPA were lower and sedentary time was greater in obese individuals, those with poor health, and those with lower educational attainment (women only). Questionnaire-derived PAEE and MVPA tended to have similar patterns of variation across socio-demographic strata. In the whole sample, domestic PA had the greatest relative contribution to total questionnaire-derived PAEE (58%), whereas occupational PA was the main driver among employed participants (54%). Only 2.2% of participants achieved an average of >30 min MVPA per day combined with >60 min strength-training per week.

**Conclusions:**

The use of both self-report and objective monitoring to assess PA in early old age provides important information on the domains of PA, PAEE and time spent at different intensity levels. Our findings suggest PA levels are generally low and observed patterns of variation indicate specific subgroups who might benefit from targeted interventions to increase PA.

## Introduction

Population ageing poses a major public health challenge owing to its impact on healthcare demands arising from chronic diseases and disabilities [1]. The ratio of workers to retired people in the EU is projected to fall from 3:1 in 2004 to 1:1 in 2050 [2]. The maintenance of good health and function with increasing age is important if older people are to be retained in the workforce. There is strong evidence that physical activity (PA) is a major modifiable health behaviour with protective effects on chronic diseases, including cardiovascular disease [3-6], type 2 diabetes [7-9], colon [10] and breast cancers [11] and osteoporosis [12]. Moreover, PA is associated with a decreased risk of cognitive decline [13], improved physical function and independent living in later life [14,15] as well as with increased life expectancy [16].

There are four principal domains in which PA can be performed; leisure, work, transportation and domestic life, which have been shown to display independent associations with health outcomes [17,18]. Due to their easy application and wide availability, questionnaires have been the most commonly used method for the assessment of PA in large-scale epidemiological studies [19]. However, subjective assessment methods may result in misclassification of PA due to social desirability bias [20], limited cognitive ability to recall frequency and duration of PA [21,22], and difficulty in accurately assigning metabolic intensity to reported activities for translation into overall PA [23]. Older individuals typically spend the majority of their time sedentary or undertaking light intensity activity [20,24-26], both of which are difficult to capture by questionnaire [20]. Furthermore, questionnaires can only estimate broad intensity categories such as sedentary, light, moderate-to-vigorous PA (MVPA), while the underlying intensity distribution is actually continuous and can only be measured with objective devices.

With an ageing global population, there is a need to comprehensively assess PA in older individuals in order to formulate public health strategies targeting lifestyle modifications in this population group. When used in combination, objective and subjective measurement methods provide complementary information on PA, i.e. accurate estimates of PA-subcomponents (i.e. PA energy expenditure (PAEE), and time spent in distinct intensity categories including MVPA, light and sedentary) assessed objectively, and self-reported information on domain, context and type of activity. A recent systematic review has highlighted that most previous studies on PA in older populations have relied exclusively on self-report [27]. Although this review concluded that there is insufficient evidence for the association between determinants and PA [27] (owing to poor methodological quality of the included studies), several studies identified male gender and good physical health as positive determinants, and age, obesity, smoking and emotional distress as negative determinants of PA [27].

Since the transition to retirement (typically age 60-64 y) is a gradual process characterised by changes in income and social networks [28], PA-patterns in this age-group are likely to be considerably different from those in younger individuals who are still fully employed as well as from those in middle old age. Therefore, this life-period offers an opportunity for interventions including PA. Only a few studies examining objectively assessed PA among individuals in later life have been conducted [25,29-33]. Those carried out in Europe had relatively small or moderately-sized samples and included participants older than 65 y (with a substantial proportion over 70 y) [32,34], who may not have been nationally representative. Therefore, their findings may not be generalisable to the population in early old age (60-64 y). Additionally, these studies examined a range of psychological, social and environmental factors in relation to accelerometer-assessed PA but did not include past history of PA.

A better understanding of the correlates of PA at different intensity levels in early old age is needed to effectively design tailored interventions. The aim of this study was to: 1) describe PA and sedentary behaviour in a nationally representative sample of British people aged 60–64, using PA-subcomponents obtained by combined heart rate (HR) and movement sensing and self-report (EPIC Physical Activity Questionnaire, EPAQ2 [35]) as complementary methods to gain a more complete view of the dimensions of PA which may not be achievable by either method alone, and 2) examine the variation of PA-subcomponents by key health- related, anthropometric and socio-demographic factors as well as prior PA collected at three previous follow-ups covering a period of over 25 y.

## Methods

### Study population

The Medical Research Council National Survey of Health and Development (NSHD) is a socially stratified birth cohort of 2547 women and 2815 men born in March 1946 across England, Scotland, and Wales who have been followed-up prospectively over 20 times since their birth [36-38].

The most recent data collection took place between 2006–2010 (at 60-64y), when 2229 (78% of eligible study members known to be alive and with a known address in England, Scotland or Wales) participated in a clinic visit at one of six clinical research facilities (N = 1690) or home visit (N = 539). Invitations were not sent to those who had died (N = 778), were living abroad (N = 570), had previously withdrawn from the study (N = 594) or had been lost to follow-up (N = 564) [38,39]. Data on many aspects of health and lifestyle were collected, including PA. The study received ethical approval from the Greater Manchester Local Research Ethics Committee and the Scotland A Research Ethics Committee, and informed consent was given by participants.

### Assessment of physical activity

#### Combined heart rate and movement sensing

At 60-64y, PA was objectively assessed using a combined heart rate (HR) and acceleration monitor (Actiheart, CamNtech Ltd, Cambridge, UK) attached to the chest with two standard ECG-electrodes [40]. All participants attending a clinic or home visit were asked to wear the monitor continuously for 5 days and given written instructions. For habitual monitoring during free-living, the combined sensor was set up for long-term monitoring to collect data in 30-second epochs. Free-living data were downloaded to a computer and robust Gaussian process regression was used to process HR for handling potential measurement noise [41].

Among those participants attending a clinic who met inclusion criteria, a step test was performed to estimate the relationship between HR and PAEE (N = 1221). Participants did not complete the step test (N = 469) if they were unwilling or, unable because they: screened positive on the Rose Angina Questionnaire [42]; reported heart disease; had ECG-abnormalities; systolic blood pressure ≥200 mmHg or diastolic blood pressure ≥120 mmHg; suffered from severe breathlessness or frequent dizziness or; had a musculoskeletal problem that could be aggravated by exercise. Those who were eligible undertook an eight-minute sub-maximal ramped step test on a 150 mm step bench [43]. The test was terminated earlier if HR reached 90% of age-predicted maximal HR [44] or was continuously above 80% of age-predicted maximal HR for 2 minutes. Those visited at home did not undertake the step test.

Individual calibration parameters were derived for those with a valid step test as described elsewhere [43]. For those without a valid step test, a group equation was derived on the basis of all valid step tests in the study (N = 1128 tests):

$$\begin{array}{ll} \mathrm{PAEE}\;\left[J/\min/\mathrm{kg}\right]= & \left(14.08-0.138\mathrm{age}+0.39\;\mathrm{sex}+0.0021\;\mathrm{SHR}\right)\\ & \left(+0.51\;\mathrm{betablocker}\right)\cdot \mathrm{HRaS}+0.94\;\mathrm{age}\\ & +5.41\;\mathrm{sex}-0.76\;\mathrm{SHR}+12.3\;\mathrm{betablocker}\\ & -84.1 \end{array}$$

(age in years, sex coded as 1 for men and 0 for women, SHR is sleeping HR in beats per minute (bpm), HRaS is HR above SHR in bpm, betablocker coded as 1 for yes and 0 for no).

Activity intensity (J/min/kg) during free-living was estimated from movement and individually calibrated HR [43] using a branched equation framework [45]. HR and movement traces from free-living for all individuals were visually inspected and if data quality was deemed to be poor, alternative models (e.g. using only acceleration) were used to estimate activity (N = 46).

Periods of non-wear were inferred from the combination of non-physiological HR and prolonged periods of inactivity, which were taken into account to minimise diurnal information bias when summarising the intensity time-series into PAEE (kJ/kg/day), average acceleration (milli-G, i.e. multiples of 0.00981 m/s^2^), and time spent in intensity levels. Measurement records with less than 48 h of valid data were excluded (N = 42). Intensity was categorised as sedentary (<1.5 MET), light (1.5-3 MET), moderate-to-vigorous (>3 MET), with 1 MET defined as 3.5 ml O_2_/kg/min [46] for all individuals. To gain a more detailed view of the PA-intensity distribution, the proportion of time spent at 19 narrowly defined intensity categories (1 to 11+ METs, with higher resolution at the lower end of spectrum but normalised to 0.25-MET category width) was also summarised and presented.

#### EPAQ2

PA was self-reported using the EPAQ2 (modified version), which assesses PA during the past year in 4 domains: leisure time PA (LTPA), occupational PA, transportation and domestic life [35]. The LTPA section of EPAQ2 is derived from the Minnesota Leisure Time Activity Questionnaire [47]. The questions on occupational PA are derived from the validated Modified Tecumseh Occupational Activity Questionnaire [48]. EPAQ2 was originally designed for use in the EPIC-Norfolk study; the version used in this study was slightly modified [49]. Transport-related PA was assessed using two questions that asked about the number and distance of journeys made by bicycle and foot to commute to and from work and get about for other reasons.

Several summary measures were derived from EPAQ2 for this study. Information on intensity of activities was obtained from the Compendium of Physical Activities [46]. Domain-specific physical activity energy expenditure (PAEE; MET-h/week) was calculated as the product of frequency, duration and intensity (in metabolic equivalent task units, MET) of each activity which was then summed across activities in each domain and across all 4 domains. Other measures were time spent at moderate-to-vigorous PA (min/day), and sedentary time (h/day). The broad intensity categories used in calculation of questionnaire-derived measures were sedentary (<1.5 MET), light (1.5-3 MET), moderate-to-vigorous (>3 MET). Sedentary time was computed from the questions capturing TV-viewing, computer use and sitting at work without differentiating week days and weekend days. We have recently compared estimates from the EPAQ2 against objective estimates in the same cohort [50].

#### Past physical activity

PA was self-reported at ages 36, 43 and 53 y during interviews with research nurses. At 36 y, participants were asked about the duration and frequency of 27 different leisure time activities during the last four weeks based on the Minnesota Leisure Time Physical Activity Questionnaire [36,47]. At 43 y, information was collected about the frequency of participation in sports, vigorous leisure activities and exercises per month and the number of months per year during which these activities were pursued. At 53 y, participants reported the number of occasions on which they participated in sports, vigorous leisure activities and exercises over the past 4 weeks. At each assessment, participants were classified into the following categories according to reported frequency of participation in relevant activities during the last four weeks/average month: inactive (no participation), moderately active (1–4 times) and most active (5 or more times).

### Assessment of other factors

#### Health status and health behaviours at 60-64 y

Body mass index (BMI, kg/m^2^) was derived from body weight and height measured using standard protocols and categorised as normal weight (<25 kg/m^2^), overweight (25–30 kg/m^2^) and obese (>30 kg/m^2^). The use of beta-blockers was self-reported and treated as a binary variable (yes/no). Participants self-reported their health status, and those reporting poor or fair health were distinguished from those reporting good, very good or excellent health. Those reporting long-term limiting illness or disability were distinguished from others. Cigarette smoking status at 60-64 y was categorised as current smoker, former smoker, or never smoker.

#### Socio-demographic factors

Occupational class at 60-64 y was categorised into 2 groups: non-manual (Registrar General social classification (RGSC) groups I, II and IIINM) and manual (RGSC groups IIIM, IV and V). Educational attainment by age 26 y was categorised into four groups: 1) degree or higher; 2) A levels, usually attained at age 18 y, or their equivalents; 3) O levels, usually attained at age 16 y, or their equivalents, or certificate of secondary education, clerical course, or equivalent; and 4) none. Employment status at 60-64 y was classified as employed full-time, employed part-time and fully retired.

#### Other factors

Season was computed based on the time of year for objective monitoring of free-living PA and used as a variable with 4 categories (spring: from April to June; summer: from July to September; autumn: from October to December, and winter: from January to March).

### Statistical analysis

Descriptive statistics for continuous variables are shown as median and corresponding interquartile range (IQR) due to departure from normal distribution or N (%) for categorical variables. To test differences in PAEE, MVPA and sedentary time across two categories or across 3 or more categories of the health-related and socio-demographic variables, Mann–Whitney U-tests and Kruskal-Wallis tests were conducted, respectively.

An *a priori* decision was made to present the results stratified by sex for comparability with previous and future studies including only men or women; formal tests for sex interactions were carried out and reported as appropriate. Multivariate tests for means were conducted to test the differences between objectively measured intensity distributions.

Median imputation was used to handle missing values for frequency and duration of individual activities from the EPAQ2, with frequency of missing data being as follows: 0.1-7.7% for the leisure time activities, 0.4-3.1% for occupational activities and 0.1-8.2% for domestic activities. Partial correlation coefficients were calculated to assess the correlation of domain-specific PAEE derived from the questionnaire with objectively measured PAEE adjusted for the other 3 domains of PA.

All analyses were performed using STATA (version 12; StataCorp, College Station TX). A double-sided p < 0.05 was considered statistically significant. When statistical interactions by sex were tested, p < 0.1 was deemed to be significant.

## Results

### Objectively assessed physical activity

Valid objective data meeting inclusion criteria were available for 1787 participants. Median (IQR) PAEE was 33.5 (25.3-42.2) kJ/kg/day and 35.5 (26.6-47.3) kJ/kg/day for women and men, respectively. Median (IQR) time spent in MVPA, light and sedentary intensities was 26.0 (12.3-48.1) min/day, 5.4 (4.2-6.7) h/day and 18.0 (16.6-19.4) h/day, respectively, among women; and 41.0 (18.8-73.0) min/day, 5.2 (4.0-6.5) h/day and 17.9 (16.3-19.4) h/day among men. Men had higher PAEE (p < 0.001) and spent more time in MVPA (p = 0.005) than women, but there was no difference in time spent in light-intensity PA or sedentary between the sexes. No significant seasonal variation in PAEE, light-intensity PA, MVPA, or sedentary time was observed in either sex. PAEE, light-intensity PA and MVPA were considerably lower among those who were obese, reported long-term limiting illness or disability or fair/poor self-rated health compared to their counterparts without these characteristics (Tables 1 and 2). MVPA was inversely associated with smoking in both sexes. However, the trend in PAEE, and light-intensity PA across the categories of smoking was significant only in women and followed the same pattern as MVPA. Fully retired participants had lower PAEE and spent less time in MVPA than those who were employed, but significance was achieved only in men. MVPA was the highest in part-time employed women and men, whereas light-intensity PA was the highest in full-time employed participants. Among the sample still working, men in manual work had higher PAEE (14%), light-intensity PA (11%) and MVPA (21%) than non-manual workers, but values for women did not substantially differ between occupational categories. In women, PAEE and MVPA were considerably greater with higher education. However, light-intensity PA did not differ by education level in women, whereas men with higher education performed less light-intensity PA than men with lower education. Individuals who reported being inactive in the past tended to have lower PAEE and MVPA than those who reported being more active at the same age, with more consistent patterns in women. Light-intensity PA, however, did not materially vary across the categories of past PA in either sex.

**Table 1:** Objectively assessed physical activity subcomponents and sedentary time in women

	**N**	**Total PAEE (kJ/kg/day)**	**Time in MVPA (min/day)**	**Time in light-intensity PA (h/day)**	**Sedentary time and sleep (h/day)**	**Acceleration (mG)**
*Total*	925	33.5 (25.3, 42.2)	26.0 (12.3, 48.1)	5.4 (4.2,6.7)	18.0 (16.6, 19.4)	8.3 (6.0, 11.0)
*Season of monitoring*						
Winter	225	33.5 (25.7, 41.0)	26.1 (10.2, 46.8)	5.4 (4.2, 6.6)	18.0 (16.7, 19.5)	8.2 (6.1, 11.0)
Spring	237	34.1 (25.3, 42.5)	28.6 (12.5, 50.3)	5.6 (4.4, 6.7)	17.9 (16.6, 19.3)	8.4 (5.8, 11.6)
Summer	244	32.8 (24.7, 41.8)	24.1 (11.7, 46.5)	5.3 (4.1, 6.8)	18.0 (16.5, 19.5)	8.4 (5.8, 10.7)
Autumn	219	33.5 (26.5, 43.3)	27.3 (13.9, 48.9)	5.4 (4.3, 6.5)	18.1 (16.7, 19.3)	8.1 (6.3, 10.7)
		p = 0.527	p = 0.519	p = 0.845	p = 0.820	p = 0.875
*Health status and health behaviours*						
BMI						
<25 kg/m^2^	300	37.0 (30.0, 47.3)*	33.3 (17.9, 62.3)	5.8 (4.8, 7.0)*	17.3 (16.1, 18.6)*	9.3 (7.0, 12.4)
25–30 kg/m^2^	336	33.7 (25.8, 41.8)	27.1 (13.8, 47.1)	5.5 (4.3, 6.6)	18.0 (16.7, 19.4)	8.4 (6.3, 11.0)
≥30 kg/m^2^	280	28.4 (21.4, 37.6)	17.2 (7.5, 37.2)	4.9 (3.8, 6.2)	18.8 (17.3, 19.4)	6.9 (4.7, 9.4)
		p < 0.001	p < 0.001	p < 0.001	p < 0.001	p < 0.001
Long term limiting illness or disability						
No	693	34.3 (26.9, 43.3)	30.4 (14.7, 52.4)	5.5 (4.4, 6.7)	17.8 (16.5, 19.1)	8.8 (6.7, 11.5)
Yes	230	28.6 (20.3, 38.1)	16.8 (4.9, 32.7)	5.0 (3.8, 6.3)	18.7 (17.1, 20.1)	6.6 (4.6, 8.9)
		p < 0.001	p < 0.001	p < 0.001	p < 0.001	p < 0.001
Self-rated health						
Excellent-Good	744	34.3 (26.5, 42.7)	29.0 (14.6, 29.6)	5.5 (4.4, 6.7)	17.8 (15.5, 19.1)	8.7 (6.5, 11.5)
Fair-Poor	108	26.1 (19.0, 36.5)	14.3 (5.6, 30.7)	4.6 (3.5, 6.4)	19.2 (17.1, 20.5)	5.7 (4.2, 8.2)
		p < 0.001	p < 0.001	p < 0.001	p < 0.001	p < 0.001
Beta-blocker use						
No	848	34.1 (26.1, 42.8)	27.6 (13.2, 49.1)	5.5 (4.4, 6.8)	17.8 (16.5, 19.2)	8.4 (6.1, 11.1)
Yes	77	24.5 (18.5, 31.6)	14.3 (6.1, 25.1)	4.0 (3.1, 5.0)	19.6 (17.6, 20.6)	7.2 (4.6, 8.7)
		p < 0.001	p < 0.001	p < 0.001	p < 0.001	p < 0.001
Smoking status						
Never	300	35.3 (27.6, 43.7)	31.3 (16.6, 53.3)	5.8 (4.6, 6.9)	17.6 (16.1, 19.0)	8.9 (6.6, 12.1)
Former	453	33.3 (25.1, 41.7)	25.6 (12.4, 46.6)	5.3 (4.0, 6.6)	18.1 (16.7, 19.6)	8.5 (6.1, 10.9)
Current	94	31.4 (23.4, 42.2)	19.7 (7.7, 43.8)	5.4 (4.2, 6.4)	18.2 (16.6, 19.5)	6.3 (4.4, 9.5)
		p = 0.003	p < 0.001	p = 0.005	p = 0.003	p < 0.001
*Sociodemographic factors*						
Employment status						
Employed full-time	104	34.1 (26.4, 41.7)	26.1 (13.9, 49.8)	5.6 (4.3, 6.7)	16.4 (14.9, 17.9)	8.1 (6.4, 10.9)
Employed part-time	240	34.8 (27.2, 44.3)	30.5 (17.2, 49.3)	5.5 (4.4, 6.7)	16.6 (15.2, 17.9)	9.0 (6.8, 11.5)
Fully retired	492	32.7 (24.4, 42.1)	24.8 (11.6, 47.6)	5.3 (4.2, 6.6)	16.9 (15.3, 18.3)	8.1 (5.7, 11.0)
		p = 0.083	p = 0.103	p = 0.281	p = 0.236	p = 0.034
Occupational class at 60-64 y (employed participants only)						
Non-manual	251	34.7 (26.8, 42.6)	29.0 (15.9, 47.6)	5.6 (4.3, 6.7)*	17.8 (16.5, 19.2)	8.8 (6.8, 11.2)
Manual	93	35.2 (27.8, 44.9)	31.2 (16.0, 59.4)	5.5 (4.7, 6.6)	17.8 (16.4, 19.0)	8.9 (6.4, 11.6)
		p = 0.501	p = 0.182	p = 0.892	p = 0.571	p = 0.871
Educational qualifications						
None	263	31.9 (23.9, 42.0)*	20.9 (9.8, 42.1)*	5.5 (4.2, 6.8)*	18.0 (16.5, 19.6)*	7.3 (5.5, 10.1)
O levels or sub GCE	318	31.5 (25.2, 41.0)	23.8 (11.8, 43.8)	5.2 (4.1, 6.5)	18.3 (16.7, 19.5)	8.0 (5.9, 10.3)
A levels	240	34.2 (27.3, 42.3)	30.4 (14.0, 53.3)	5.5 (4.4, 6.6)	17.8 (16.6, 19.1)	8.9 (6.7, 12.2)
Degree or higher	56	36.7 (30.2, 44.2)	47.1 (25.9, 61.4)	5.6 (4.6, 6.4)	17.5 (16.6, 19.8)	9.6 (7.8, 13.1)
		p = 0.012	p < 0.001	p = 0.582	p = 0.242	p < 0.001
*Physical activity at*						
36 y						
Most active	316	34.8 (26.9, 43.4)	30.2 (15.5, 53.8)	5.5 (4.4, 6.7)	17.9 (16.5, 19.3)	8.6 (6.6, 11.9)
Less active	222	33.8 (25.7, 42.2)	27.9 (14.2, 47.8)	5.4 (4.4, 6.6)	18.0 (16.6, 19.2)	8.3 (6.1, 10.8)
Inactive	311	31.2 (23.4, 39.8)	19.6 (8.1, 40.5)	5.2 (4.0, 6.6)	18.2 (16.7, 19.7)	7.6 (5.5, 10.0)
		p = 0.005	p < 0.001	p = 0.443	p = 0.092	p < 0.001
43 y						
Most active	206	34.6 (27.5, 43.6)	35.2 (16.5, 58.3)	5.5 (4.4, 6.6)	17.7 (16.6, 19.0)	9.3 (6.9, 12.4)
Less active	225	33.7 (25.8, 43.6)	29.3 (14.3, 59.4)	5.4 (4.4, 6.4)	18.0 (16.6, 19.2)	8.6 (6.6, 11.9)
Inactive	454	32.3 (23.5, 40.6)	22.9 (9.8, 42.9)	5.5 (4.1, 6.9)	18.0 (16.5, 19.6)	7.8 (5.6, 10.0)
		p = 0.007	p < 0.001	p = 0.879	p = 0.378	p < 0.001
53 y						
Most active	325	34.8 (27.9, 43.4)	32.1 (16.1, 59.6)	5.6 (4.4, 6.6)	17.7 (16.5, 19.0)	9.3 (7.0, 12.7)
Less active	171	34.0 (26.0, 44.0)	25.4 (11.8, 49.6)	5.5 (4.4, 6.8)	17.8 (16.6, 19.1)	8.4 (6.4, 11.2)
Inactive	402	31.4 (23.2, 39.9)	21.3 (9.9, 41.2)	5.3 (4.0, 6.6)	18.3 (16.7, 19.7)	7.4 (5.5, 9.7)
		p < 0.001	p < 0.001	p = 0.048	p = 0.004	p < 0.001

*Abbreviations:* PAEE- physical activity energy expenditure, MVPA- moderate-to-vigorous physical activity; Data are median (IQR); MVPA was defined as intensity >3 MET, light-intensity PA as intensity between 1.5-3.0 MET and sedentary behaviour as intensity <1.5 MET; Total N varies due to variation in the amount of missing data for different covariates; *p < 0.1 for interaction by sex.

**Table 2:** Objectively assessed physical activity subcomponents and sedentary time in men

	**N**	**Total PAEE (kJ/kg/day)**	**Time in MVPA (min/day)**	**Time in light-intensity PA (h/day)**	**Sedentary time and sleep (h/day)**	**Acceleration (mG)**
*Total*	862	35.5 (26.6, 47.3)	41.0 (18.8, 73.0)	5.2 (4.0, 6.5)	17.9 (16.3, 19.4)	8.6 (6.3, 11.6)
*Season of monitoring*						
Winter	205	32.9 (24.2, 43.3)	36.5 (15.6, 64.5)	5.1 (4.0, 6.0)	18.2 (16.8, 19.6)	7.9 (5.7, 10.4)
Spring	223	36.6 (28.0, 49.8)	41.5 (20.4, 87.0)	5.5 (4.2, 7.0)	17.7 (15.8, 19.0)	9.0 (6.8, 12.6)
Summer	248	35.5 (27.4, 48.2)	41.8 (19.9, 70.3)	5.3 (4.0, 6.8)	18.0 (15.9, 19.4)	9.1 (6.4, 11.6)
Autumn	186	36.3 (27.5, 47.5)	42.9 (18.6, 78.1)	5.2 (4.0, 6.5)	17.9 (16.4, 19.4)	8.5 (6.3, 11.7)
		p = 0.066	p = 0.263	p = 0.080	p = 0.048	p = 0.008
*Health status and health behaviours*						
BMI						
<25 kg/m^2^	231	37.5 (28.3, 49.8)	48.7 (20.8, 90.2)	5.3 (4.0, 6.5)	17.7 (16.2, 19.2)	9.2 (0.07, 0.12)
25–30 kg/m^2^	392	36.8 (28.8, 47.3)	47.3 (22.1, 74.0)	5.4 (4.2, 6.6)	17.7 (16.1, 19.2)	9.2 (0.07, 0.12)
≥30 kg/m^2^	236	29.9 (23.3, 43.4)	28.7 (13.2, 54.2)	5.0 (3.8, 6.8)	18.5 (16.5, 19.8)	7.2 (0.05, 0.10)
		p < 0.001	p < 0.001	p = 0.096	p = 0.006	p < 0.001
Long term limiting illness or disability						
No	667	37.0 (28.7, 49.3)	47.2 (22.8, 80.7)	5.4 (4.2, 6.7)	17.7 (16.0, 19.2)	9.2 (6.8, 12.2)
Yes	193	29.5 (20.8, 40.8)	24.9 (10.3, 49.4)	4.7 (3.3, 6.0)	18.9 (17.2, 20.3)	6.8 (4.6, 9.1)
		p < 0.001	p < 0.001	p < 0.001	p < 0.001	p < 0.001
Self-rated health						
Excellent-Good	672	37.0 (28.8, 48.7)	46.2 (23.1, 77.2)	5.4 (4.2, 6.7)	17.7 (16.0, 19.2)	9.1 (6.8, 12.1)
Fair-Poor	110	27.7 (20.0, 37.7)	20.8 (10.3, 45.3)	4.5 (3.1, 5.8)	19.1 (17.6, 20.4)	6.1 (4.2, 8.2)
		p < 0.001	p < 0.001	p < 0.001	p < 0.001	p < 0.001
Beta-blocker use						
No	757	36.6 (28.0, 48.4)	44.0 (20.4, 77.2)	5.4 (4.2, 6.6)	17.8 (16.1, 19.2)	8.8 (6.4, 11.8)
Yes	81	26.9 (18.8, 34.3)	23.4 (7.4, 39.7)	4.2 (3.1, 5.1)	19.6 (18.3, 20.7)	6.9 (4.6, 9.4)
		p < 0.001	p < 0.001	p < 0.001	p < 0.001	p < 0.001
Smoking status						
Never	235	34.5 (27.1, 46.0)	44.2 (20.5, 77.2)	5.1 (4.1, 6.7)	18.1 (16.3, 19.3)	8.8 (6.1, 11.7)
Former	473	36.9 (27.4, 48.5)	43.7 (21.4, 76.2)	5.3 (4.1, 6.5)	17.8 (16.1, 19.4)	8.7 (6.5, 12.0)
Current	77	35.0 (24.5, 44.4)	30.6 (9.4, 58.8)	5.4 (3.9, 6.8)	17.9 (16.1, 19.7)	8.1 (5.6, 10.3)
		p = 0.301	p = 0.006	p = 0.977	p = 0.710	p = 0.056
*Sociodemographic factors*						
Employment status						
Employed full-time	394	36.8 (28.0, 48.3)	43.4 (20.3, 75.3)	5.4 (4.0, 6.8)	17.7 (16.0, 19.3)	8.8 (6.7, 11.9)
Employed part-time	125	37.2 (30.7, 51.7)	55.0 (27.3, 87.9)	5.1 (4.1, 6.5)	18.0 (15.7, 19.1)	9.7 (6.8, 12.4)
Fully retired	252	33.4 (25.0, 43.1)	36.8 (16.8, 65.2)	5.1 (4.0, 6.1)	18.1 (16.7, 19.6)	7.7 (5.5, 10.6)
		p = 0.004	p = 0.006	p = 0.164	p = 0.097	p < 0.001
Occupational class at 60-64 y (employed participants only)						
Non-manual	307	35.8 (27.7, 47.6)	41.3 (21.3, 71.2)	5.1 (4.0, 6.5)	18.1 (16.3, 19.5)	9.0 (6.7, 11.7)
Manual	211	40.8 (29.3, 53.3)	49.8 (20.8, 89.3)	5.7 (4.5, 7.2)	17.1 (15.8, 19.0)	9.2 (6.9, 12.6)
		p = 0.008	p = 0.177	p = 0.002	p = 0.002	p = 0.502
Educational qualifications						
None	264	36.5 (26.4, 49.3)	40.2 (14.7, 75.3)	5.5 (4.2, 7.1)	17.7 (15.6, 19.3)	8.4 (5.7, 11.9)
O levels or sub GCE	167	35.2 (26.4, 46.0)	41.3 (19.2, 79.6)	5.1 (4.0, 6.3)	17.9 (16.3, 19.4)	8.6 (6.2, 11.4)
A levels	248	35.8 (27.7, 47.3)	40.2 (19.8, 72.5)	5.3 (4.0, 6.5)	17.9 (16.4, 19.4)	8.7 (6.3, 11.5)
Degree or higher	137	36.2 (27.8, 45.3)	49.1 (23.6, 77.2)	5.0 (4.0, 6.3)	18.2 (16.5, 19.5)	9.2 (6.9, 12.0)
		p = 0.834	p = 0.534	p = 0.046	p = 0.226	p = 0.432
*Physical activity at*						
36 y						
Most active	331	35.4 (26.9, 48.5)	45.2 (20.5, 83.7)	5.2 (4.0, 6.3)	18.0 (16.4, 19.4)	9.3 (6.8, 12.5)
Less active	233	36.5 (26.9, 48.5)	40.1 (18.8, 65.7)	5.5 (4.1, 7.2)	17.8 (15.8, 19.3)	8.2 (6.1, 10.5)
Inactive	231	34.8 (26.0, 44.6)	38.7 (17.0, 68.7)	5.1 (4.0, 6.4)	18.1 (16.4, 19.4)	8.1 (5.8, 10.8)
		p = 0.349	p = 0.160	p = 0.061	p = 0.140	p < 0.001
43 y						
Most active	254	38.1 (28.7, 51.4)	51.1 (24.8, 88.3)	5.3 (4.2, 6.6)	17.7 (16.1, 19.3)	9.8 (6.8, 13.1)
Less active	202	34.7 (25.8, 47.5)	36.9 (17.7, 69.1)	5.4 (4.0, 6.7)	18.0 (16.2, 19.5)	8.9 (7.8, 11.5)
Inactive	362	34.9 (26.2, 45.4)	38.9 (17.0, 67.7)	5.2 (4.0, 6.5)	18.0 (16.3, 19.4)	8.1 (5.9, 10.4)
		p = 0.025	p = 0.002	p = 0.803	p = 0.400	p < 0.001
53 y						
Most active	283	37.0 (26.9, 50.4)	47.4 (23.1, 80.6)	5.2 (4.0, 6.3)	17.8 (16.1, 19.4)	9.2 (7.4, 12.2)
Less active	188	37.9 (27.6, 47.6)	48.1 (25.2, 79.9)	5.4 (4.4, 6.6)	17.5 (16.2, 18.9)	9.5 (6.9, 12.3)
Inactive	338	33.9 (25.2, 45.1)	35.6 (15.0, 65.2)	5.3 (4.0, 6.8)	18.1 (16.3, 19.4)	7.8 (5.8, 10.7)
		p = 0.010	p < 0.001	p = 0.401	p = 0.178	p < 0.001

*Abbreviations:* PAEE- physical activity energy expenditure, MVPA- moderate-to-vigorous physical activity Data are median (IQR); MVPA was defined as intensity >3.0 MET, light-intensity PA as intensity between 1.5-3.0 MET and sedentary behaviour as intensity <1.5 MET; Total N varies due to variation in the amount of missing data for different covariates.

Generally, sedentary time followed a reverse trend of that observed for PAEE, light-intensity PA and MVPA. Obese individuals, those with long-term limiting illness or disability, poor/fair self-rated health and fully retired participants spent more time sedentary than their respective counterparts (Tables 1 and 2). Among the sample still working, sedentary time was substantially lower in men in manual work compared with those in non-manual work, but did not significantly differ between occupational categories among women. Opposing trends in sedentary time in women and men were found across the categories of education (interaction by sex: p = 0.013), whereby women with higher educational qualifications had lower sedentary time than less educated women, whereas the reverse was seen in men. Those who were inactive in the past tended to spend more time sedentary than those who reported being active at the same age, with weaker trends in men. In women, beta-blocker users had lower PAEE (28%) and MVPA (48%) and spent more time sedentary (10%) than non-users, with similar differences in men. Additionally, after adjustment for age and season, PAEE, MVPA and sedentary time did not differ between testing sites in England, Scotland and Wales (data not shown).

Median (IQR) acceleration was 8.3 (6.0-11.0) mG and 8.6 (6.3-11.6) mG among women and men respectively, and followed similar patterns of association with covariates as PAEE.

**Figure 1:**
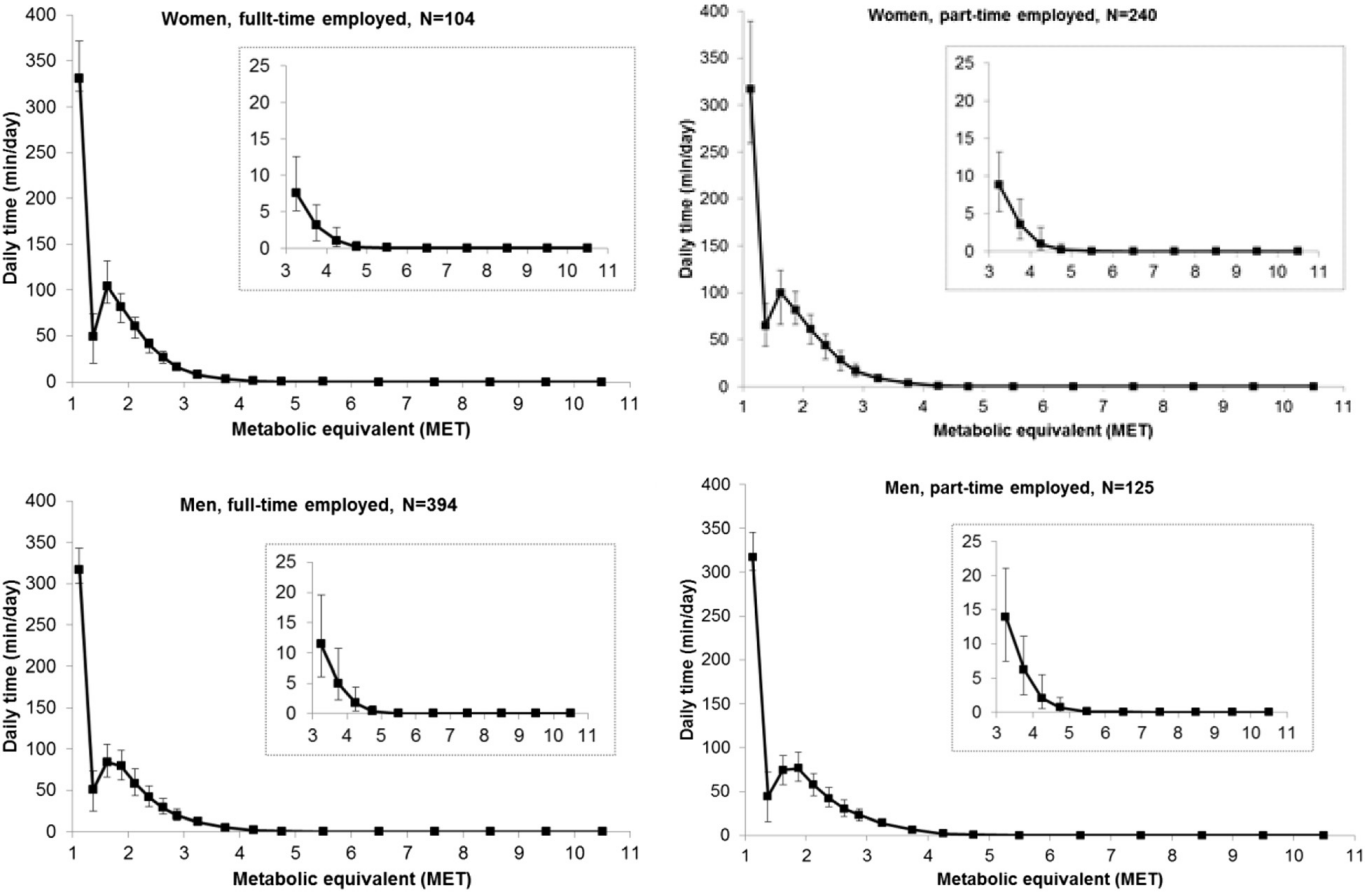
**Physical activity intensity distributions, stratified by sex for employed (manual and non-manual workers) and retired participants at age 60–64 years.** Values are median (IQR) daily durations (min/day). Time spent at 1 MET (not plotted) among women was 648 (590, 720) min/day, 676 (605, 763) min/day and 691 (619, 763) min/day for non-manual workers, manual workers and retired, respectively, whilst the same estimates in men were 677 (619, 763) min/day, 662 (590, 734) min/day and 705 (634, 792) min/day. Inserts of each graph show zoomed view of intensity distribution in the MVPA (>3 METs) zone. All values have been normalised to bin size 0.25 METs.

Participants spent a median (IQR) of 68% (60%-75%) of total PAEE in the light-intensity category (1.5-3 MET). Figure 1 shows the detailed activity intensity distributions by sex for manual and non-manual workers and for retired participants. Intensity distributions were similar in all 6 sex- and occupation/employment strata, with approximately 75% of daily time spent below 1.5 MET (70% below 1.25 MET, 5% at 1.25-1.5 MET) and 12% at 1.5-2.0 MET. Time spent in the higher intensity categories was generally much lower than that spent at lower intensities, although a slightly greater proportion of time was spent in 1.50-1.75 MET than in 1.25-1.50 MET which corresponds to light PA. Time spent at 4–4.5 MET (moderate PA) approached 0 and virtually no time was spent at intensities higher than that. For example, in non-manual working women, time spent at 1–1.25 MET was 331 (274, 339) min/day and at 1.25-1.5 MET 61 (42, 93) min/day. This dropped to 16 (10, 24) min/day at 3–3.5 MET which equates to 8 (5, 12) min/day when normalised to 0.25-MET category width and had reached 0 for intensities >6 MET. According to the multivariate test for means, there was a significant difference between intensity distributions of manual and non-manual workers in men (p < 0.001) but not in women (p = 0.585). Furthermore, we tested the difference in intensity distributions between fully retired individuals and both employed groups (non-manual and manual including both part-time and full-time employed) for men and women separately and found no significant differences. Also, we did not observe substantial differences in intensity distribution between full-time and part-time employed participants (p = 0.437 and p = 0.131 among women and men, respectively; Figure 1). To confirm whether the observed overall non-significant differences in intensity distributions were driven by near-zero (non-variant) time spent in vigorous PA, we performed separate sensitivity analyses excluding the categories ≥6 MET; differences between non-manual and manual workers were still non-significant in women, but remained significant in men.

### Self-reported physical activity

Median (IQR) PAEE was 18.2 (13.0, 24.5) MET-h/day and 19.6 (12.8, 28.1) MET-h/day, for women and men, respectively. Median (IQR) MVPA, light-intensity PA and awake sedentary time was 63.0 (29.5-117.8) min/day, 4.1 (2.2, 5.6) h/day and 4.0 (2.0-5.0) h/day among women; and 87.5 (43.6-167.0) min/day, 2.0 (1.1, 3.4) h/day and 4.6 (3.1-6.4) h/day among men. Women had lower PAEE (p = 0.004), participated in less MVPA but were less sedentary and performed more light-intensity PA than men (all p < 0.001).

Trends in PAEE and time in MVPA across the categories of BMI, long-term limiting illness or disability, self-rated health, employment status, occupational class, past PA and smoking were consistent with the trends in objectively assessed variables (Tables 3 and 4). PAEE from EPAQ2 was higher in full-time employed participants than in those who were employed part-time or retired, whereas MVPA was higher in part-time employed individuals (Tables 3 and 4) as also observed for objective measures. Among men, PAEE and time in MVPA were materially greater in those in manual than non-manual occupations, but in women, this difference was not significant. Women with higher educational qualifications had higher PAEE and MVPA than those with lower education but the opposite patterns were observed for men (interaction by sex: p = 0.019 for PAEE, p = 0.054 for MVPA). Light-intensity PA from EPAQ2 generally followed the trends observed for the corresponding objectively measured light PA variable. However, self-reported light-intensity PA did not significantly differ by BMI-category. In addition, there was an inverse relationship between education and light-intensity PA in women and men. Women classified as inactive in the past reported spending considerably more time in light-intensity PA than those classified being more active in the past.

**Table 3:** Self-reported physical activity subcomponents and sedentary time in women

	**N**	**Total PAEE (MET-h/day)**	**N**	**Time in MVPA (min/day)**	**N**	**Time in light-intensity PA (h/day)**	**N**	**Sedentary time (h/day)**
*Total*	1143	18.2 (13.0, 24.5)	1162	62.0 (29.5, 117.8)	1162	4.1 (2.2, 5.6)	1162	4.0 (2.0, 5.0)
*Health status and health behaviours*								
BMI								
<25 kg/m^2^	369	19.4 (14.2, 25.9)	376	80.0 (38.9, 136.5)	376	4.0 (2.8, 5.4)	376	3.0 (2.5, 4.5)
25–30 kg/m^2^	418	18.4 (13.7, 24.8)	425	62.4 (35.3, 119.4)	425	4.3 (3.2, 5.8)	425	4.0 (3.0, 5.0)
≥30 kg/m^2^	337	16.1 (11.8, 22.9)	341	49.1 (17.6, 94.8)	341	4.1 (3.0, 5.7)	341	4.3 (3.0, 5.0)
		p < 0.001		p < 0.001		p = 0.253		p < 0.001
Long term limiting illness or disability								
No	842	19.4 (14.0, 25.5)	855	71.2 (36.1, 128.4)	855	4.2 (3.1, 5.6)	855	3.7 (2.9, 5.0)*
Yes	294	14.7 (10.1, 20.5)	299	44.9 (12.1, 87.7)	299	3.9 (2.4, 5.6)	299	4.0 (3.0, 4.5)
		p < 0.001		p < 0.001		p = 0.005		p = 0.320
Self-rated health								
Excellent-Good	915	18.8 (13.7, 25.3)*	928	68.9 (35.5, 124.8)	928	4.1 (3.0, 5.6)	928	3.9 (2.9, 5.0)
Fair-Poor	137	13.9 (9.2, 19.4)	139	36.2 (11.0, 80.8)	139	4.0 (2.4, 5.9)	139	4.0 (3.0,4.5)
		p < 0.001		p = 0.017		p = 0.221		p = 0.027
Beta-blocker use								
No	901	18.4 (13.6, 24.8)	911	68.0 (32.5, 123.1)	911	4.2 (3.0, 5.6)	911	4.0 (3.0, 5.0)
Yes	82	17.3 (11.2, 22.8)	84	46.8 (20.6, 91.9)		3.7 (2.9, 6.0)	84	3.5 (2.7, 4.5)
		p = 0.041		p = 0.009		p = 0.464		p = 0.365
Smoking status								
Never	371	18.3 (13.7, 24.7)	378	64.0 (32.5, 123.3)	378	4.2 (3.9, 5.5)	378	3.5 (2.5, 4.5)
Former	558	18.4 (13.2, 25.5)	566	67.7 (32.5, 120.4)	566	4.1 (3.0, 5.6)	566	4.0 (3.0, 5.0)
Current	115	16.7 (11.9, 22.3)	117	43.1 (17.5, 102.5)	117	4.1 (3.1, 5.8)	117	4.5 (3.0, 5.0)
		p = 0.100		p = 0.456		p = 0.929		p = 0.006
*Sociodemographic factors*								
Employment status								
Employed full-time	128	22.1 (16.8, 28.5)*	130	61.3 (37.4, 136.3)	130	4.3 (3.1, 5.9)	130	5.3 (32.7-7.5)
Employed part-time	280	20.8 (15.3, 27.5)	284	81.9 (40.2, 133.3)	284	4.4 (3.2, 5.8)	284	4.0 (2.9, 5.1)
Fully retired	619	16.3 (11.8, 22.0)	627	59.3 (27.2, 107.5)	627	3.9 (2.9, 3.4)	627	3.5 (2.5, 4.5)
		p < 0.001		p = 0.087		p < 0.001		p < 0.001
Occupational class at 60-64 y (employed participants only)								
Non-manual	298	21.1 (16.3, 27.3)*	303	75.6 (38.9, 131.3)	303	4.3 (3.2, 5.7)	303	4.4 (3.0, 6.1)
Manual	109	21.2 (14.8, 28.5)	110	82.6 (37.1, 148.7)	110	4.9 (3.2, 6.4)	110	4.0 (3.0, 6.0)
		p = 0.979		p = 0.339		p = 0.227		p = 0.137
Educational qualifications								
None	329	16.9 (12.2, 23.4)*	340	44.6 (18.1, 102.6)*	340	4.6 (3.3, 6.5)	340	4.0 (3.0, 4.5)*
O levels or sub GCE	395	18.8 (13.3, 24.8)	400	62.1 (31.0, 111.1)	400	4.2 (3.1, 5.8)	400	4.0 (3.0, 5.0)
A levels	293	17.8 (13.3, 23.5)	295	75.2 (41.4, 127.6)	295	3.8 (2.8, 4.9)	295	3.5 (2.7, 4.5)
Degree or higher	68	20.2 (13.8, 25.1)	68	(50.7, 131.8)	68	4.7)		4.0)
		p = 0.139		p < 0.001		p < 0.001		p < 0.001
*Physical activity at*								
36 y								
Most active	376	19.2 (14.1, 25.7)	386	80.8 (44.2, 131.5)	386	3.9 (2.9, 5.6)	386	3.6 (2.5, 5.0)*
Less active	274	18.8 (13.5, 25.5)	277	64.3 (35.3, 121.5)	277	4.1 (3.0, 5.6)	277	4.0 (3.0, 4.7)
Inactive	401	16.4 (11.6, 22.4)	406	42.4 (16.2, 93.8)	406	4.3 (3.1, 5.8)	406	4.0 (3.0, 5.0)
		p < 0.001		p = 0.471		p = 0.045		p = 0.281
43 y								
Most active	253	19.3 (14.6, 25.4)	257	85.5 (48.0, 140.7)	257	3.9 (2.9, 5.5)	257	3.5 (2.6, 4.5)
Less active	268	18.4 (13.1, 24.8)	272	65.1 (37.1, 122.2)	272	4.0 (2.8, 5.5)	272	4.0 (2.8, 5.0)
Inactive	572	17.5 (12.2, 24.1)	582	51.6 (20.6, 102.9)	582	4.3 (3.2, 5.9)	582	4.0 (3.0, 5.0)
		p < 0.001		p = 0.090		p = 0.016		p = 0.031
53 y								
Most active	396	19.5 (14.1, 26.7)*	511	84.7 (44.3, 138.0)	511	3.9 (2.9, 5.4)	511	3.5 (2.5, 5.0)
Less active	203	17.8 (13.2, 24.8)	206	65.3 (36.2, 118.8)	206	4.2 (3.2, 5.3)	206	4.0 (3.0, 5.0)
Inactive	504	16.7 (12.0, 22.4)	404	45.8 (17.4, 87.7)	404	4.3 (3.1, 6.0)	404	4.0 (3.0, 5.0)
		p < 0.001		p = 0.230		p = 0.019		p = 0.090

*Abbreviations:* PAEE- physical activity energy expenditure, MVPA- moderate-to-vigorous physical activity; Data are median (IQR); MVPA was defined as intensity >3 MET, light-intensity PA as intensity between 1.5-3.0 MET and sedentary behaviour as intensity <1.5 MET; Total N denotes the number of participants with valid data for the PA-subcomponent in the subsequent column and varies due to variation in the amount of missing data for different covariates; *p < 0.1 for interaction by sex.

**Table 4:** Self-reported physical activity subcomponents and sedentary time in men

	**N**	**Total PAEE (MET-h/day)**	**N**	**Time in MVPA (min/day)**	**N**	**Time in light-intensity PA (h/day)**	**N**	**Sedentary time (h/day)**
*Total*	1046	19.6 (12.8, 28.1)	1059	87.5 (43.6, 167.0)	1059	2.0 (1.1, 3.4)	1061	4.6 (3.1, 6.4)
*Health status and health behaviours*								
BMI								
<25 kg/m^2^	261	21.2 (13.6, 28.3)	265	89.5 (46.9, 169.5)	265	1.9 (1.0, 3.1)	265	4.5 (3.0, 6.0)
25–30 kg/m^2^	481	20.6 (13.4, 28.8)	486	96.1 (48.8, 184.7)	486	2.0 (1.1, 3.5)	487	4.5 (3.0, 6.3)
≥30 kg/m^2^	288	18.1 (11.0, 26.6)	292	74.7 (32.1, 144.9)	292	2.1 (1.3, 3.4)	292	4.8 (3.5, 7.1)
		p = 0.026		p = 0.004		p = 0.272		p = 0.020
Long term limiting illness or disability								
No	789	20.9 (14.1, 28.9)	799	97.8 (50.9, 179.1)	799	2.0 (1.2, 3.4)	802	5.0 (3.3, 7.0)
Yes	249	15.5 (7.8, 25.3)	252	62.4 (18.9, 125.0)	252	2.0 (1.0, 3.2)	251	4.5 (3.0, 5.7)
		p < 0.001		p < 0.001		p = 0.282		p < 0.001
Self-rated health								
Excellent-Good	810	20.8 (13.9, 28.5)	819	95.8 (51.8, 172.4)	819	2.1 (1.2, 3.4)	821	4.8 (3.3, 6.9)
Fair-Poor	140	13.2 (6.5, 19.7)	142	46.2 (9.2, 108.8)	142	1.9 (0.9, 3.0)	142	4.5 (3.0, 5.0)
		p < 0.001		p < 0.001		p = 0.061		p = 0.144
Beta-blocker use								
No	811	20.5 (13.5, 28.8)	818	89.9 (47.3, 173.2)	818	2.0 (1.2, 3.3)	820	4.6 (3.1, 6.6)
Yes	91	15.3 (10.2, 23.9)	93	79.0 (33.6, 128.0)	93	2.1 (1.0, 3.1)	93	4.5 (3.5, 6.3)
		p = 0.004		p = 0.008		p = 0.934		p = 0.708
Smoking status								
Never	273	19.5 (13.4, 26.9)	274	96.7 (45.7, 166.8)	274	2.1 (1.0, 3.3)	275	4.6 (3.0, 6.3)
Former	572	20.0 (12.9, 28.5)	580	86.9 (48.3, 169.3)	580	2.0 (1.2, 3.3)	581	4.7 (3.4, 6.7)
Current	109	17.8 (10.5, 27.7)	111	71.9 (24.8, 150.3)	111	2.3 (1.1, 3.4)	111	4.5 (3.0, 6.0)
		p = 0.324		p = 0.446		p = 0.507		p = 983
*Sociodemographic factors*								
Employment status								
Employed full-time	456	22.6 (16.1, 30.1)	472	94.5 (50.9, 187.6)	472	2.1 (1.2, 3.7)	471	5.7 (3.7, 8.0)
Employed part-time	152	21.4 (14.5, 28.5)	153	113.8 (62.5, 178.9)	153	2.1 (1.4, 3.1)	153	4.7 (3.5, 6.0)
Fully retired	316	13.9 (8.2, 22.2)	322	68.9 (29.0, 130.6)	322	1.7 (0.9, 3.1)	322	4.0 (2.9, 5.0)
		p < 0.001		p < 0.001		p = 0.007		p < 0.001
Occupational class at 60-64 y (employed participants only)								
Non-manual	363	20.8 (15.2, 28.0)	367	80.7 (49.6, 141.4)	367	2.1 (1.2, 3.5)	367	6.0 (4.1, 7.9)
Manual	252	24.3 (17.0, 34.2)	255	142.1 (65.1, 271.2)	255	2.1 (1.3, 3.6)	255	4.5 (3.3, 6.3)
		p < 0.001		p < 0.001		p = 0.728		p = 0.002
Educational qualifications								
None	322	21.5 (11.8, 29.8)	330	101.4 (38.0, 200.2)	330	2.3 (1.4, 3.9)	327	4.5 (3.3, 6.0)
O levels or sub GCE	204	19.4 (11.8, 28.2)	206	90.4 (36.5, 167.3)	206	2.0 (1.0, 3.6)	204	4.7 (3.0, 6.4)
A levels	297	19.2 (13.2, 27.5)	300	83.9 (44.8, 158.4)	300	2.0 (1.2, 3.2)	299	4.5 (3.0, 6.3)
Degree or higher	168	18.3 (13.3, 24.6)	168	75.9 (49.3, 129.3)	168	1.7 (1.0, 2.5)	168	5.0 (3.6, 7.4)
		p = 0.383		p < 0.001		p < 0.001		p < 0.001
*Physical activity at:*								
36 y								
Most active	402	21.5 (14.2, 29.0)	410	106.5 (57.3, 174.6)	410	1.9 (1.0, 3.1)	410	4.7 (3.1, 6.6)
Less active	269	19.1 (12.0, 27.0)	271	73.8 (39.1, 156.8)	271	2.2 (1.2, 3.4)	271	4.7 (3.2, 6.9)
Inactive	281	18.4 (11.4, 27.3)	286	72.9 (34.5, 161.0)	286	3.6)	286	4.5 (3.1, 6.0)
		p = 0.009		p = 0.023		p = 0.354		p = 0.595
43 y								
Most active	306	21.6 (15.4, 29.5)	311	109.7 (59.5, 109.7)	311	1.8 (1.0, 3.0)	311	5.0 (3.4, 7.1)
Less active	242	19.7 (12.4, 23.5)	244	92.4 (52.0, 152.6)	244	2.0 (11, 3.3)	244	4.5 (6.0, 6.5)
Inactive	438	18.3 (12.0, 27.9)	444	72.8 (34.5, 169.3)	444	2.1 (1.2, 3.5)	444	4.5 (3.0, 6.0)
		p < 0.001		p = 0.197		p = 0.054		p = 0.177
53 y								
Most active	348	22.2 (14.9, 31.7)	354	113.0 (37.3, 185.6)	354	1.8 (1.1, 3.1)	354	5.0 (3.5, 6.7)
Less active	207	22.2 (14.4, 28.9)	208	102.5 (57.3, 198.1)	208	2.0 (1.2, 3.0)	208	4.5 (3.0, 7.1)
Inactive	421	17.4 (10.2, 25.3)	428	56.8 (25.3, 129.9)	428	2.1 (1.2, 3.7)	428	4.5 (3.0, 6.0)
		p < 0.001		p = 0.343		p = 0.079		p = 0.189

*Abbreviations:* PAEE- physical activity energy expenditure, MVPA- moderate-to-vigorous physical activity; Data are median (IQR); MVPA was defined as intensity >3 MET, light-intensity PA as intensity between 1.5-3.0 MET and sedentary behaviour as intensity <1.5 MET; N denotes the number of participants with valid data for the PA-subcomponent in the subsequent column and varies due to variation in the amount of missing data for different covariates.

Self-reported sedentary time followed the same pattern as objectively recorded time with respect to obesity, self-rated health, long-term limiting illness or disability, smoking and occupational class (Tables 3 and 4). In contrast to objectively assessed total sedentary time which was the highest in fully retired participants, self-reported awake sedentary time was the highest among full-time employed individuals in both sexes.

### Domain-specific physical activity

In the entire sample, total questionnaire-derived PAEE was driven by domestic activities with median (IQR) of 8.1 (4.8-12.2) MET-h/day (Table 5). However, when the analysis was limited to employed participants (approximately 40% of women and 66% of men), the relative contribution of occupational PA to the total PAEE indicated that work was the predominant domain, with median (IQR) of 11.0 (4.8, 16.6) MET-h/day. Median (IQR) reported total occupational activity was 30.0 (14.0-40.1) h/week, the majority of which was spent sitting and doing light activities. Partial correlations between domain-specific PAEE and total objectively assessed PAEE are shown in Table 5. After adjustment for all other domains, there was a weak positive and significant correlation between all domains and total objectively assessed PAEE, with highest correlations for LTPA (r = 0.155, p < 0.001) and occupational PA (r = 0.134, p < 0.001). In the sub-sample including only employed participants, the strength of these correlations were lower for LTPA (r = 0.126, p < 0.001) and occupational PA (r = 0.075, p = 0.033 respectively). Transport-related PA had the smallest contribution to total PAEE from the questionnaire (4% in the whole sample and 6% among the employed) but was still weakly positively associated with total PAEE from combined sensing among all participants. We conducted several sensitivity analyses to further examine domain-specific PAEE with respect to employment status and occupational class and observed higher LTPA in part-time employed and fully retired participants compared with full-time employed individuals. Total PAEE from the questionnaire was predominantly driven by domestic PA (58%) in all participants and by occupational PA (54%) in the employed participants. Stratification by occupational class showed that the contribution of occupational PA to the total questionnaire-derived PAEE was 53% in non-manual and 56% in manual workers with similar contributions of other domains in both occupational classes.

**Table 5:** Self-reported domain-specific physical activity and partial correlation with objectively measured total physical activity energy expenditure

**All (N = 1705)**			
Domain	PAEE (MET-h/day)	r	p-value for r
Leisure	1.7 (0.4, 3.9)	0.155	<0.001
Occupational	1.5 (0, 11.6)	0.134	<0.001
Transport-related	2.3 (0.5, 5.4)	0.126	<0.001
Domestic	8.1 (4.8, 12.2)	0.077	0.002
Total	16.0 (11.0, 22.5)	-	-
**Employed (N = 819)**			
Domain			
Leisure	1.6 (0.4, 3.8)	0.126	<0.001
Occupational	11.0 (4.8, 16.6)	0.075	0.033
Transport-related	2.5 (0.6, 6.0)	0.063	0.072
Domestic	6.7 (4.0, 10.8)	0.062	0.075
Total	19.2 (13.9, 26.1)	-	-

*Abbreviation:* PAEE- physical activity energy expenditure.

r- partial correlation coefficient (adjusted for all other domains).

Data are median (IQR). Among non-employed participants, PAEE in occupational domain was 0.

## Discussion

### Principal findings

In a nationally representative sample of British adults aged 60-64y we found evidence that PAEE, MVPA, light-intensity PA and sedentary time vary by sex, past PA, health status and behaviours, and socio-demographic parameters, and that patterns of variation are similar when either objective or self-reported instruments are used to assess PA. Overall, median (IQR) objectively measured MVPA was 26.0 (12.3-48.1) and 41.0 (18.8, 73.0) min/day in women and men, respectively.

Both PAEE and time spent in MVPA were greater among men than women. Self-reported sedentary time was greater among men, but objective estimates indicated that there was no difference between genders. Obese individuals and those with fair/poor self-rated health had lower PAEE and MVPA, and longer sedentary time compared with their counterparts. Employed participants had greater PAEE and more time in MVPA but less sedentary time (objectively measured) than retired people. Those who self-reported being active in the past had greater PAEE and time in MVPA and were less sedentary than those reporting less activity in the past. Objective estimates indicated that participants spent 75% of their time at the intensity below 1.5 MET (sedentary). Domestic PA had the greatest relative contribution to the total self-report PAEE (MET-h/day), whereas among the employed, total self-reported PAEE was mainly driven by occupational PA.

### Comparison with other studies

Research examining PA in older populations has mainly relied on self-report measures [17,51-53] and only a few epidemiological studies have used objective methods specifically with older adults [20,34,54]. However, none of the previous studies using objective methods examined detailed PA-patterning according to various socio-demographic, behavioural or clinical parameters. The observed differences in PAEE and MVPA by sex are plausible and in keeping with the findings of previous studies which used questionnaires [49,55,56] or objective methods [54,34] to assess PA.

The substantially lower self-reported sedentary time in women than men is consistent with previous studies [25]. However, the discrepancy in sex-differences by method (non-significant difference in objectively measured sedentary time between the sexes) might be explained by the gender-specific sedentary pursuits; it is possible that not all sedentary pursuits in which women would normally engage were captured by the EPAQ2, and women did therefore not have the opportunity to report such activities. Another possible explanation would be a differential reporting bias of sedentary time, i.e. greater underestimation among women [50].

Parsons *et al.*[49] used the similar modified version of the questionnaire in the 1958 British birth cohort who were aged 45 at the time of assessment and still in the workforce. In this other birth cohort higher PAEE was reported than in our population which supports the notion that PA declines with age [20,51]. Further, our observation that retired persons had greater PAEE during leisure time (as ascertained by self-report) than their full-time employed counterparts is in line with a recent systematic review indicating that LTPA and exercise increase after the transition to retirement [28]. In contrast, total objectively assessed PAEE in our study was lower in retired participants compared with full-time employed individuals, which suggests a redistribution of PA from the occupational domain to leisure time, but also an overall decrease in PA after retirement. Higher LTPA in part-time employed relative to full-time employed participants is consistent with some studies [57], but contrary to Parsons *et al.*[49] who reported a positive association between working hours and LTPA in men. This may reflect the fact that our sample of part-time employed participants included those who had retired from their main occupation but continued to work in a different occupation. Besson *et al.*[17] reported EPAQ2 results from the EPIC-Norfolk population which was of similar age when the questionnaire was administered (1998–2000) as the population in the present study (2006–2010) and found comparable levels of total and domain-specific PAEE.

Manini e*t al.*[58] assessed PAEE in a slightly older population [mean age 74.8 (±2.9) y, N = 302, 50% women] using the doubly labelled water technique and reported mean PAEE of 672 kcal/day. Mean PAEE in our population was 36.2 kJ/kg/day which equals 680 kcal/day (mean body weight was 78.9 kg). This suggests similar PAEE in these populations; however differences in assessment methods and population sampling procedures need to be considered. Objectively assessed levels of PAEE and MVPA in our study are lower than those observed in the InterAct study where the same method was used in 54-y old adults from 10 European countries (median PAEE of 40.5 and 44.0 kJ/kg/day in women and men, respectively; and median MVPA-time of 72.5 and 80.8 min/day in women and men, respectively) [56]. Troiano *et al.*[20] reported that accumulated mean time in MVPA assessed by accelerometer (>2020 counts per min, ~3 MET) in the NHANES- population was 12.4 min/day in women and 16.7 min/day in men aged 60–69. However, when only MVPA occurring in 10-min bouts or longer was included, these estimates were as low as 5.8 and 6.5 min/day in women and men, respectively [20]. Both total accumulated and bout-based estimates are considerably lower than those observed in our study. Davis *et al*. [34] examined PA by accelerometry in 163 European women and men (Better Ageing project), aged over 70 and reported a mean PAEE estimate of 16.8 and 20.1 kJ/kg/day in women and men, respectively and mean MVPA of 16.7 and 23.8 min/day in women and men, respectively. The observed differences may reflect different methods used to assess PA or unequal wear time and period of day during which the monitor was worn. In the NHANES and the Better Ageing project, a uniaxial accelerometer was worn around the waist during awake hours over 7 days [20], whereas in our study, a combined HR and acceleration sensor was worn for 5 days. In addition, hip-worn accelerometers do not capture activities such as cycling or upper body movement well, whereas the combined sensing performs better at distinguishing intensity across most activities.

With respect to intensity distribution, our finding that over 75% of the time was spent below 1.5 MET, is congruent with the results of Evenson *et al.*[54] suggesting that adults aged 60–69 spend most of their awake time sedentary. Mean sedentary time during awake hours in the NHANES was 617 and 569 min/day in women and men, respectively [20]. Assuming 8 h of sleep, these findings are comparable to ours. Lower PAEE and less time in MVPA in current smokers than non-smokers is in keeping with the results of the EPIC-Norfolk [59] cohort showing a greater proportion of inactive adults among current smokers, which highlights the fact that unhealthy behaviours are likely to cluster and suggests the need to promote PA as part of general lifestyle modification advice in this population subgroup.

Several other studies have examined the patterning of objectively measured PA by socio-demographic factors. Cleland *et al.*[60] indicated that Australian adults (mean age 31 y) with manual occupation and lower education had 5.8 times higher pedometer-assessed PA than their counterparts. However, it is difficult to directly attribute the observed differences to age alone, given the different methods used to assess PA. The inverse relationship of BMI with PAEE and MVPA is in line with our previous findings from the EPIC-Norfolk cohort which showed a significant cross-sectional relationship between body weight and inactivity, and demonstrated that weight gain over time is associated with future physical inactivity [61]. Although BMI is an indicator of overall body composition, it does not distinguish between fat and fat-free mass, but PA in this age-group has been found to play a major role in the preservation of fat-free mass [62-64].

### Strengths and limitations

The major strength of our study lies in the combined use of objective and self-reported measures to obtain complementary information on PA. Objective monitoring gives estimates of total free-living PAEE and time spent at different intensity levels, whereas questionnaire data provide valuable information on the type and context of activity and allow an estimation of domain-specific PAEE and contribution of different domains to total PAEE. In addition, our sample is unique as it is nationally representative of British adults aged 60–64 at the time of assessment [39], which is a population of particular public health interest given the changes in lifestyle and health occurring with transition to retirement. Moreover, we examined the patterns of time spent in light-intensity PA which adds to the existing knowledge of PA in this population and provides important information for the design of PA-interventions tailored for people in later life. Lastly, the birth cohort design allowed us to explore the variation of PA-measures by past PA, independent of the confounding effect of age.

However, important caveats should be considered in the interpretation of our findings. Firstly, objectively assessed sedentary behaviour includes sleep, which did not allow us to make conclusions about the amount of time spent sedentary during awake hours. Secondly, the possibility of social desirability bias in self-reported PA and sedentary time, as well as bias arising from assigning energy cost to these behaviours [46], cannot be excluded. In addition, the possibility of reverse causality and/or reporting bias cannot be ruled out in cross-sectional associations with health-related factors including BMI, long-term limiting illness or disability, and self-rated health.

The interpretation of how our findings relate to current UK-guidelines for PA [65] is not straight-forward. Since we did not have a full week of free-living PA-monitoring, we cannot accurately assess total weekly duration of MVPA on which the guidelines are based (≥150 min/week). If we extrapolate our objectively measured MVPA-estimates per day into weekly values assuming that MVPA is pursued *all* days of the week, the findings broadly suggest that 55% of women and 69% of men in our sample meet UK-guidelines for MVPA (≥150 min/week). If however, we use the common interpretation of the guidelines that MVPA should exceed 30 min/day, then our results indicate that 43% of women and 60% of men meet the recommended level. Furthermore, current guidelines also state that adults should perform muscle-strengthening activities at least twice a week. If we count the individuals who report doing ≥1 h/week of weight-training on top of accumulating ≥30 min/day of objectively measured MVPA, the proportion of participants meeting this combined target is only 2.2% (1.8% among women and 2.4% among men) indicating a low prevalence of sufficient activity. This proportion would be even lower if any further restrictions on MVPA time accumulated in bouts (e.g. lasting 10 min or longer) were imposed.

### Possible implications

This study demonstrates the utility of the combined use of self-report and objective monitoring to assess the levels and context of PA in early old age. Our findings indicate a detrimental cross-sectional relationship of poor health with PA which may in turn lead to reduced independent living and loss of function. Promoting PA earlier in adulthood and maintaining it in later life should be considered as the means to achieve health benefits and reduce the growing healthcare spending associated with deteriorating health in older individuals. Since PAEE and time in MVPA were materially lower among retired participants compared with the employed, encouragement to substantially increase LTPA needs to be given to retired adults. Given the robust evidence that physical inactivity, overweight and obesity are risk factors for exit from paid employment via disability pension [66,67], particularly in older workers [67], PA-promotion in the working population should be considered as a primary preventive effort to reduce premature exit from the labour market and improve sustainable work ability, as well as establishing healthy habits before retirement. A recent meta-analysis has suggested that PA-promotion in this age group has some effect on increasing PA [68]. Our findings highlight specific population strata which could be targeted for such PA-promotion efforts and further demonstrate that individuals in this age-group spend a large proportion of their time in light-intensity PA (1.5-3 MET). The variation within the light PA category warrants further research to clarify potential health benefits.

## Conclusions

In conclusion, the patterns of PAEE, MVPA, light-intensity PA and sedentary time in early old age differ between the sexes and across several socio-demographic, clinical and behavioural factors. Our study suggests that several modifiable factors (e.g., BMI, smoking) should be taken into account in designing interventions aiming to increase PA and reduce sedentary time. Because early old age is a critical life period marked by transition from working life to retirement, the parameters related to work, as well as other domains of life, should be comprehensively evaluated and considered as potential correlates of PA.

## Competing interests

The authors declare that they have no competing interests.

## Authors’ contributions

RG, KM, DK, RC, and SB conceived the study. RG and SB analysed the data and RG wrote the first draft of the manuscript. All authors contributed to the critical revision of all manuscript drafts and approved the final version of the paper.
